# Common Polymorphisms Influencing Serum Uric Acid Levels Contribute to Susceptibility to Gout, but Not to Coronary Artery Disease

**DOI:** 10.1371/journal.pone.0007729

**Published:** 2009-11-05

**Authors:** Klaus Stark, Wibke Reinhard, Martina Grassl, Jeanette Erdmann, Heribert Schunkert, Thomas Illig, Christian Hengstenberg

**Affiliations:** 1 Klinik und Poliklinik für Innere Medizin II, Universitätsklinikum Regensburg, Regensburg, Germany; 2 Medizinische Klinik II, Universitätsklinikum Schleswig-Holstein - Campus Lübeck, Lübeck, Germany; 3 Institute of Epidemiology, HelmholtzZentrum München, München-Neuherberg, Germany; New York University School of Medicine, United States of America

## Abstract

**Background:**

Recently, a large meta-analysis including over 28,000 participants identified nine different loci with association to serum uric acid (UA) levels. Since elevated serum UA levels potentially cause gout and are a possible risk factor for coronary artery disease (CAD) and myocardial infarction (MI), we performed two large case-control association analyses with participants from the German MI Family Study. In the first study, we assessed the association of the qualitative trait gout and ten single nucleotide polymorphisms (SNP) markers that showed association to UA serum levels. In the second study, the same genetic polymorphisms were analyzed for association with CAD.

**Methods and Findings:**

A total of 683 patients suffering from gout and 1,563 healthy controls from the German MI Family Study were genotyped. Nine SNPs were identified from a recently performed genome-wide meta-analysis on serum UA levels (rs12129861, rs780094, rs734553, rs2231142, rs742132, rs1183201, rs12356193, rs17300741 and rs505802). Additionally, the marker rs6855911 was included which has been associated with gout in our cohort in a previous study. SNPs rs734553 and rs6855911, located in *SLC2A9*, and SNP rs2231142, known to be a missense polymorphism in *ABCG2*, were associated with gout (*p* = 5.6*10^−7^, *p* = 1.1*10^−7^, and *p* = 1.3*10^−3^, respectively). Other SNPs in the genes *PDZK1*, *GCKR*, *LRRC16A*, *SLC17A1*-*SLC17A3*, *SLC16A9*, *SLC22A11* and *SLC22A12* failed the significance level. None of the ten markers were associated with risk to CAD in our study sample of 1,473 CAD cases and 1,241 CAD-free controls.

**Conclusion:**

SNP markers in SLC2A9 and ABCG2 genes were found to be strongly associated with the phenotype gout. However, not all SNP markers influencing serum UA levels were also directly associated with the clinical manifestation of gout in our study sample. In addition, none of these SNPs showed association with the risk to CAD in the German MI Family Study.

## Introduction

Gout is mainly caused by elevated serum uric acid (UA) levels [1]. Several studies showed significant association between single nucleotide polymorphism (SNP) markers in *SLC2A9* gene (solute carrier family 2, member 9, also known as *GLUT9* gene) and serum UA levels as well as susceptibility to gout [2]–[6]. Additionally, Dehghgan et al. reported association between markers in genes *ABCG2* and *SLC17A3* with both, serum UA levels and gout in a large cohort [6]. Very recently, Kolz et al. conducted a meta-analysis of 14 genome-wide association (GWA) studies on serum UA levels including a total of 28,141 participants [7]. Nine loci with significant associations to serum UA levels were found, namely the genes *PDZK1*, *GCKR*, *LRRC16A*, *SLC16A9* and *SLC22A11* together with the previously reported findings in *ABCG2, SLC2A9* and *SLC17A1*-*SLC17A3*, as well as the intensively studied *SLC22A12* gene encoding for URAT1. Therefore, five novel loci associated with serum UA levels emerged from this meta-analysis [7]. The advantage of this GWA-based meta-analysis is its power to detect novel common variants with relatively small phenotypic effects on serum UA due to the large sample size.

We analyzed these new and known loci for their association with the clinical phenotype gout in a case control study.

Elevated serum UA levels are potentially increasing the risk for coronary artery disease (CAD) and myocardial infarction (MI) [8]–[10]. We therefore tested additionally for the influence of these SNP markers on the susceptibility to CAD in our German MI Family Study.

## Materials and Methods

### Ethics Statement

The Ethics committee of the University of Regensburg approved the study protocol and all participants gave their written informed consent at the time of inclusion and again at the time of follow-up investigations. The study was in accordance with the principles of the current version of the Declaration of Helsinki.

### Case-Control Samples and Phenotyping

All individuals of this study participated in the German MI Family Study (total *n* = 7,575). Recruitment process, selection criteria and study details have been reported previously [3]. A total of *n* = 683 unrelated individuals (*n* = 480 males, *n* = 203 females) with the diagnosis of gout were selected from the German MI Family Study. Phenotyping was carried out as reported previously [3]. In brief, the phenotype gout was established using medical history readings and self-reported history of gout.

Controls (*n* = 1,563) were unrelated individuals from our German MI Family Study who neither had any indication of gout nor were they medicated with uricostatic or uricosuric agents at any time during follow-up (*n* = 871 males, *n* = 692 females). Phenotypic details are shown in Table 1.

**Table 1:** Characteristics of gout case and control study sample.

Variable	Gout cases (*n* = 683)	Gout-free controls (*n* = 1,563)	*p*-value
Gender, % male (*n*)	70.3 (480)	55.7 (871)	<0.0001
Age, years (range) a	58.3±9.5 (23–84)	58.5±8.6 (28–87)	n. s.
Medication with diuretics, % (*n*)	36.1 (221)	22.0 (341)	<0.0001
MI or severe CAD, % (*n*)	61.1 (417)	58.2 (909)	n. s.
Hypercholesterolemia b, % (*n*)	70.5 (481)	66.9 (1,046)	n. s.
Lipid lowering medication, % (*n*)	50.1 (307)	44.9 (701)	0.03
LDL-C, mg/dl	150.9±41.0	147.8±38.7	n. s.
HDL-C, mg/dl	50.7±14.2	55.3±15.7	<0.0001
Hypertension c, % (*n*)	86.6 (580)	83.7 (1,269)	0.05
Antihypertensive therapy, % (*n*)	83.5 (512)	72.6 (1,134)	<0.0001
Systolic blood pressure, mmHg	139.0±19.1	135.8±18.5	0.0003
Diastolic blood pressure, mmHg	84.0±10.3	82.0±9.8	<0.0001
Type 2 diabetes d, % (*n*)	16.3 (111)	10.6 (165)	0.0002
Smoking e, % (*n*)	66.1 (451)	60.4 (942)	0.009
BMI, kg/m^2^	28.1±3.9	26.8±3.7	<0.0001

Values denote means±standard deviations unless indicated otherwise. n. s., not significant; CAD, coronary artery disease; MI, myocardial infarction; LDL-C, low-density lipoprotein cholesterol; HDL-C, high-density lipoprotein cholesterol; BMI, body mass index.

a At inclusion to study.

b Defined as LDL-C ≥160 mg/dL or intake of lipid lowering medication.

c Defined as blood pressure ≥140/90 mmHg or ongoing antihypertensive therapy.

d Defined as history of diabetes mellitus or intake of antidiabetic medication.

e Former or current smoking habit.

Furthermore, a large case-control sample was established from the German MI Family Study including *n* = 1,473 CAD/MI unrelated cases (*n* = 856 males, *n* = 617 females) and *n* = 1,241 unrelated CAD/MI-free control individuals (*n* = 336 males, *n* = 905 females). MI was diagnosed according to MONICA (Monitoring Trends and Determinants in Cardiovascular Disease) diagnostic criteria (http://www.ktl.fi/publications/monica/manual/index.htm). Severe CAD was defined as prior MI, treatment with percutaneous coronary intervention or coronary artery bypass graft. Cardiovascular risk factors and phenotypic details are summarized in Table 2.

**Table 2:** Characteristics of CAD case and control study sample.

Variable	CAD cases (*n* = 1,473)	CAD-free controls (*n* = 1,241)	*p*-value
Gender, % male (*n*)	58.1 (856)	27.1 (336)	<0.0001
Age at inclusion, years (range)	60.2±8.5 (32–90)	56.4±9.9 (29–84)	<0.0001
Age at first CAD event, years (range)	54.5±9.1 (24–89)	-	-
MI, % (*n*)	75.6 (1,114)	-	-
Gout, % (*n*)	15.5 (228)	8.8 (109)	<0.0001
Hypercholesterolemia a, % (*n*)	83.4 (1,228)	29.3 (363)	<0.0001
Lipid lowering medication, % (*n*)	66.7 (982)	38.2 (474)	<0.0001
LDL-C, mg/dl	149.4±42.6	146.1±34.9	0.0313
HDL-C, mg/dl	51.4±13.8	61.6±15.3	<0.0001
Hypertension b, % (*n*)	94.4 (1,390)	53.9 (669)	<0.0001
Antihypertensive therapy, % (*n*)	89.3 (1,316)	35.0 (434)	<0.0001
Systolic blood pressure, mmHg	140.0±20.4	132.6±18.2	<0.0001
Diastolic blood pressure, mmHg	82.6±10.4	81.4±9.8	0.0054
Type 2 diabetes c, % (*n*)	11.6 (171)	4.2 (52)	<0.0001
Smoking d, % (*n*)	62.7 (924)	48.1 (597)	<0.0001
BMI, kg/m^2^	27.3±3.6	26.5±4.2	<0.0001

Values denote means±standard deviations unless indicated otherwise. CAD, coronary artery disease; MI, myocardial infarction; LDL-C, low-density lipoprotein cholesterol; HDL-C, high-density lipoprotein cholesterol; BMI, body mass index.

a Defined as LDL-C ≥160 mg/dL or intake of lipid lowering medication.

b Defined as blood pressure ≥140/90 mmHg or ongoing antihypertensive therapy.

c Defined as history of diabetes mellitus or intake of antidiabetic medication.

d Former or current smoking habit.

Recent GWA analyses [11], [12] using a part of the current study sample (*n* = 1,021) revealed no population stratification effects within unrelated individuals form the German MI Family Study using the genomic control method [13]. Therefore, no correction for population stratification was carried out.

### SNP Selection and Genetic Analyses

Genomic DNA isolation using the PureGene DNA Blood Kit (Qiagen, Hilden, Germany) and genotyping with 5′ exonuclease TaqMan® technology (Applied Biosystems, Foster City, CA, USA) was carried out as previously described [3]. SNPs were selected from a recently published meta-analysis on serum UA levels [7] and our previous study [3].

### Statistical Analyses

To determine whether the SNP genotypes of cases and controls deviated from Hardy-Weinberg equilibrium (HWE), actual and predicted genotype counts of both groups were compared by χ^2^-test. Differences in allele frequencies between dichotomous traits were calculated employing the same method. Prevalence odds ratios (OR) with their 95% confidence intervals (CI) were reported. Continuous parameters were compared by *t* test for normally distributed values or otherwise by non-parametric tests. Logistic regression was used to adjust for covariates differentially distributed in case-control cohorts. Full adjustment model for gout included gender, medication with diuretics, lipid lowering and antihypertensive therapy, high-density lipoprotein cholesterol (HDL-C), type 2 diabetes, smoking, and BMI. The corresponding model for CAD case-control sample included gender, age at inclusion, hypercholesterolemia, hypertension, type 2 diabetes, smoking, and BMI. Employing a model based on allele dosage, epistasis between SNPs was tested using a logistic regression analysis with the second SNP as a covariate. A two-sided *p*-value≤0.05 was considered statistically significant.

Statistical and association analyses were performed using JMP 7.0.2 (SAS Institute Inc, Cary, NC, USA) and PLINK v1.06 [14], respectively. Power analysis was carried out using G*Power 3.0.10 employing a two-tailed exact test with minor allele frequencies (MAF) from controls [15].

## Results

### Population Characteristics

In our first case-control cohort, gout cases (*n* = 683) were compared to control individuals (*n* = 1,563). Prevalence of cardiovascular risk factors and cardiovascular disease was high in both, gout cases and gout-free controls. However, we found no significant difference in number of reported MI or CAD events between gout cases (61.1% CAD/MI) and gout-free controls (58.2% CAD/MI). The proportion of women was lower in the gout group than in the control group. Gout cases were more often treated with diuretics as compared to controls. In addition and in concordance to the clinical manifestation of gout cases, the prevalence of type 2 diabetes and increased body mass index (BMI) was higher, and gout-free controls showed higher HDL-C levels, even after adjusting for gender (*p*<0.001). The prevalence of hypercholesterolemia was equally distributed between the two groups, whereas hypertension and smoking were slightly more prevalent in gout cases (Table 1).

In our second, large case-control sample for CAD/MI the incidence of established cardiovascular risk factors, such as male gender, type 2 diabetes, hypercholesterolemia, hypertension and smoking, as well as increased BMI, was higher in CAD/MI cases (*n* = 1,473) as compared to controls (*n* = 1,241) (Table 2). We also found more individuals suffering from gout in our CAD/MI cases compared to CAD/MI-free controls (Table 2).

### Genetic Analyses

The cohorts were genotyped for markers listed in Table 3. All SNPs fulfilled our criteria of at least 98% call rate in all sub-samples, except for rs734553 with a total call rate = 96.0%. Marker rs6855911 is in strong LD with rs734553 (*r^2^* = 0.94) and, therefore, can to some degree be used as a surrogate. Data for rs734553 were reported for completeness. Strong LD (*r^2^* = 0.967) exits between rs1183201 (*SLC17A1*) reported from Kolz et al. [7] and rs1165205 (*SLC17A3*) described by Dehghan et al. [6]. Therefore, a distinction between these two genes on association level is not possible.

**Table 3:** SNP marker used in analysis.

SNP	Position a	Major allele (1)	Minor allele (2)	Gene name(s)	Function	Call rate b
rs12129861	Chr1: 144,437,046	G	A	*PDZK1*	5′ Intergenic	98.4%
rs780094	Chr2: 27,594,741	C	T	*GCKR*	Intron 16	99.2%
rs734553	Chr4: 9,532,102	T	G	*SLC2A9 GLUT9*	Intron 7	96.0%
rs6855911	Chr4: 9,545,008	A	G	*SLC2A9 GLUT9*	Intron 7	99.0%
rs2231142	Chr4: 89,271,347	G	T	*ABCG2*	Exon 5 Q141K	99.2%
rs742132	Chr6: 25,715,550	A	G	*LRRC16A*	Intron 34	99.4%
rs1183201	Chr6: 25,931,423	T	A	*SLC17A1*	Intron 3	98.2%
rs12356193	Chr10: 61,083,359	A	G	*SLC16A9*	Intron 5	98.7%
rs17300741	Chr11: 64,088,038	G	A	*SLC22A11*	Intron 4	98.2%
rs505802	Chr11: 64,113,648	T	C	*SLC22A12 URAT1*	5′ Intergenic	99.3%

a on human genome build 18.

b in total sample (*n* = 4,960).

#### Association analysis of SNPs in the gout case-control sample

Genotype distributions and allele frequencies in gout case-control cohort are shown in Table 4. No deviation from HWE was observed for the ten genotyped markers in gout-free controls (*p*>0.23). However, as previously reported [3], rs6855911 in *SLC2A9* gene showed deviation from HWE in gout cases (*p* = 0.01). The proximate marker rs734553 also showed nominal deviation from HWE in gout cases (*p* = 0.05), whereas the other markers exhibited *p*-values>0.18. Significant association with gout was found for rs734553 and rs6855911 located in intron 7 of *SLC2A9*, even after correction for multiple testing (ten SNPs) with *p_corr_* = 5.6*10^−6^ and *p_corr_* = 1.1*10^−6^, respectively. The *ABCG2* polymorphism rs2231142 remained significantly associated with gout after correction for multiple testing with *p_corr_* = 0.013. The power to detect nominal association with *p* = 0.05 and OR = 1.2 for the other SNPs ranged from 32.8% to 50.4% (Table 4). Interaction between SNPs was analyzed using a model based on allele dosage. Nominal significance was observed between SNPs in *SLC2A9* (rs734553 and rs6855911) and rs742132 in *LRRC16A* with *p* = 0.038 and *p* = 0.024, respectively.

**Table 4:** Association analysis results in gout case-control sample.

	Gout case genotypes				Gout-free control genotypes				Allelic	Allelic OR	Adjusted a	Power b	Power b
SNP	11	12	22	MAF	11	12	22	MAF	*p*-value	(95% CI)	*p*-value	OR = 1.2	OR = 1.4
rs12129861	187	334	149	0.472	394	752	394	0.500	0.083	0.89 (0.78–1.02)	0.090	0.504	0.953
rs780094	240	325	112	0.406	558	747	247	0.400	0.723	1.02 (0.90–1.17)	0.472	0.490	0.950
rs734553	429	211	14	0.183	846	553	103	0.253	5.6*10^−7^	0.66 (0.56–0.78)	3.7*10^−6^	0.418	0.909
rs6855911	429	233	15	0.194	829	603	114	0.269	1.1*10^−7^	0.66 (0.56–0.77)	1.3*10^−6^	0.430	0.918
rs2231142	500	168	9	0.137	1,241	299	12	0.104	1.3*10^−3^	1.37 (1.13–1.66)	2.2*10^−3^	0.239	0.663
rs742132	330	276	73	0.311	764	644	145	0.301	0.502	1.05 (0.91–1.20)	0.996	0.452	0.932
rs1183201	187	320	161	0.481	393	791	354	0.487	0.679	0.97 (0.86–1.11)	0.691	0.504	0.953
rs12356193	475	182	14	0.157	1,069	436	40	0.167	0.385	0.93 (0.78–1.10)	0.427	0.328	0.819
rs17300741	176	337	158	0.487	409	770	355	0.482	0.798	1.02 (0.89–1.16)	0.981	0.504	0.953
rs505802	317	298	64	0.314	721	682	148	0.315	0.917	0.99 (0.87–1.14)	0.783	0.459	0.936

Numbers of genotypes (11, 12, 22) according to alleles from Table 3.

a Model including gender, medication with diuretics, lipid lowering and antihypertensive therapy, HDL-C, type 2 diabetes, smoking, and BMI.

b Power was calculated for the given OR using the respective MAF in controls and a two-tailed *p* = 0.05.

Furthermore, we had indication for gender interaction, as separate analyses in females and males revealed association with gout for both *SLC2A9* SNPs, whereas *ABCG2* SNP rs2231142 only showed significant association in males (Table 5), but not in females (Table 6). Full adjustment for gender, medication with diuretics, lipid lowering and antihypertensive therapy, HDL-C, type 2 diabetes, smoking, and BMI did not did not change the association results substantially (Table 4). The same model without inclusion of gender was applied in males and females separately and did not lead to a significant change in *p*-values (Table 5 and Table 6, respectively).

**Table 5:** Association analysis results in male gout case-control sample.

	Gout case genotypes				Gout-free control genotypes				Allelic	Allelic OR	Adjusted a
SNP	11	12	22	MAF	11	12	22	MAF	*p*-value	(95% CI)	*p*-value
rs12129861	135	232	104	0.467	218	415	228	0.506	0.056	0.86 (0.73–1.00)	0.145
rs780094	164	225	87	0.419	315	411	138	0.398	0.277	1.09 (0.93–1.28)	0.293
rs734553	289	159	12	0.199	453	318	64	0.267	1.1*10^−4^	0.68 (0.56–0.83)	6.0*10^−4^
rs6855911	291	174	12	0.208	445	349	69	0.282	2.2*10^−5^	0.67 (0.55–0.80)	3.0*10^−4^
rs2231142	345	124	7	0.145	686	172	7	0.108	4.4*10^−3^	1.41 (1.11–1.78)	3.3*10^−3^
rs742132	224	203	50	0.318	438	354	73	0.289	0.122	1.15 (0.96–1.36)	0.511
rs1183201	126	232	109	0.482	230	440	188	0.476	0.757	1.03 (0.87–1.20)	0.991
rs12356193	334	127	9	0.154	586	252	22	0.172	0.237	0.88 (0.71–1.09)	0.334
rs17300741	116	236	120	0.504	228	426	199	0.483	0.295	1.09 (0.93–1.28)	0.421
rs505802	215	214	48	0.325	386	384	92	0.330	0.812	0.98 (0.83–1.16)	0.781

Numbers of genotypes (11, 12, 22) according to alleles from Table 3.

a Model including medication with diuretics, lipid lowering and antihypertensive therapy, HDL-C, type 2 diabetes, smoking, and BMI.

**Table 6:** Association analysis results in female gout case-control sample.

	Gout case genotypes				Gout-free control genotypes				Allelic	Allelic OR	Adjusted a
SNP	11	12	22	MAF	11	12	22	MAF	*p*-value	(95% CI)	*p*-value
rs12129861	52	102	45	0.482	176	337	166	0.493	0.720	0.96 (0.77–1.20)	0.315
rs780094	76	100	25	0.373	243	336	109	0.403	0.288	0.88 (0.70–1.11)	0.734
rs734553	140	52	2	0.144	393	235	39	0.235	1.4*10^−4^	0.55 (0.40–0.75)	1.3*10^−3^
rs6855911	138	59	3	0.163	384	254	45	0.252	2.0*10^−4^	0.58 (0.43–0.77)	9.7*10^−4^
rs2231142	155	44	2	0.119	555	127	5	0.100	0.256	1.22 (0.86–1.74)	0.265
rs742132	106	73	23	0.295	326	290	72	0.315	0.426	0.91 (0.71–1.16)	0.391
rs1183201	61	88	52	0.478	163	351	166	0.502	0.386	0.91 (0.73–1.13)	0.507
rs12356193	141	55	5	0.162	483	184	18	0.161	0.958	1.01 (0.75–1.36)	0.778
rs17300741	60	101	38	0.445	181	344	156	0.482	0.194	0.86 (0.69–1.08)	0.270
rs505802	102	84	16	0.287	335	298	56	0.298	0.687	0.95 (0.74–1.21)	0.923

Numbers of genotypes (11, 12, 22) according to alleles from Table 3.

a Model including medication with diuretics, lipid lowering and antihypertensive therapy, HDL-C, type 2 diabetes, smoking, and BMI.

#### Association analysis of SNPs in the CAD/MI case-control sample

Deviation from HWE was not observed for the genotyped markers in CAD/MI cases and CAD/MI-free controls (*p*>0.05). No association with CAD was found for any of the analyzed SNPs (Table 7). Again, adjustment for differentially distributed risk factors between CAD cases and controls did not alter the results significantly (Table 7). The power to detect nominal association with CAD was >30.3% for an assumed OR = 1.2 and >79.6% for an OR = 1.4 (Table 7).

**Table 7:** Association analysis results in CAD case-control sample.

	CAD case genotypes				CAD-free control genotypes				Allelic	Allelic OR	Adjusted a	Power b	Power b
SNP	11	12	22	MAF	11	12	22	MAF	*p*-value	(95% CI)	*p*-value	OR = 1.2	OR = 1.4
rs12129861	379	712	355	0.492	329	605	288	0.483	0.537	1.04 (0.93–1.15)	0.785	0.655	0.992
rs780094	539	691	228	0.393	433	603	198	0.405	0.393	0.95 (0.85–1.06)	0.125	0.639	0.991
rs734553	817	505	89	0.242	726	404	59	0.220	0.055	1.14 (1.00–1.29)	0.984	0.509	0.964
rs2231142	1,140	305	16	0.115	991	226	13	0.102	0.131	1.14 (0.96–1.36)	0.055	0.303	0.796
rs742132	715	607	137	0.302	606	495	129	0.306	0.740	0.98 (0.87–1.10)	0.543	0.593	0.985
rs1183201	385	743	321	0.478	320	608	290	0.488	0.477	0.96 (0.86–1.07)	0.638	0.655	0.992
rs12356193	1,026	389	36	0.159	848	353	29	0.167	0.417	0.94 (0.81–1.09)	0.065	0.432	0.926
rs17300741	385	686	368	0.494	336	315	273	0.474	0.149	1.08 (0.97–1.21)	0.745	0.655	0.992
rs505802	681	641	136	0.313	595	520	115	0.305	0.516	1.04 (0.93–1.17)	0.996	0.592	0.984

Numbers of genotypes (11, 12, 22) according to alleles from Table 3.

a Model including age at inclusion, gender, hypercholesterolemia, diabetes, hypertension, smoking, and BMI.

b Power was calculated for the given OR using the respective MAF in controls and a two-tailed *p* = 0.05.

## Discussion

In the present case-control association studies, we evaluated the relationship of common SNPs with gout and their potential influence on CAD. The variants are located in nine different genetic regions, four of which are known and the remaining five loci were only recently identified to be associated with serum UA levels in a large meta-analysis of GWA studies [7]. We were able to confirm significant association between gout and SNPs in two established genes, namely *SLC2A9* (rs734553 and rs6855911) and *ABCG2* (rs2231142). However, for markers in the other known and novel loci, no association with the clinical phenotype gout was found in our study. Moreover, our results indicate no relevant influence of the investigated polymorphisms on CAD susceptibility in our German MI Family Study.

The strongest association signal with gout was detected for intronic SNPs rs6855911 and rs734553 in the *SLC2A9* gene, which is consistent with previous studies on gout and serum UA levels [2]–[6]. *SLC2A9* is coding for GLUT9, a high-capacity urate transporter, which is abundantly expressed in liver and kidney tissues [16]–[18]. It is noteworthy that both SNPs located in *SLC2A9* gene showed deviation from HWE in gout cases, which can in some degree support a true association [19], [20].

In addition, we found a significant association between the exonic SNP rs2231142 in *ABCG2* and gout, again supporting the results of a prior GWA on serum UA levels and gout [6]. This is the only marker examined that leads to a missense mutation with an amino acid exchange from glutamine to lysine at position 141 in ABCG2 transporter and therefore could have a direct and causal influence on development of the disease [6]. It is notable, however, that the effect of this variant on susceptibility to gout is only present in our male subcohort.

A recent meta-analysis documented an additional locus on chromosome 6p23-p21.3 encompassing three members of the solute carrier family 17 (SLC17A1, SLC17A3 and SLC17A4) to be associated with serum UA levels [7]. Interestingly, their top marker rs1183201 in the *SLC17A1* gene did not show significant association with the qualitative trait of gout in our study. Another SNP marker, rs1165205 in *SLC17A3*, which is in strong LD with rs1183201 in *SLC17A1* was previously found to be related to serum UA levels and also representing a risk factor for gout [6]. A possible explanation for these discrepancies may lie in the different recruitment strategies of the study populations and the distinct definition of the phenotype “gout”. While Kolz et al. [7] in their meta-analysis examined participants of European ancestry from 14 different study cohorts with widely varying initial inclusion criteria – potentially concealing a substructure which could lead to false positive results – our ascertainment approach was to recruit individuals with a strong familial history of CAD from all over Germany with a concomitant accumulation of cardiometabolic risk factors, such as gout. On the other hand, Deghan et al. [6] included participants from three large population-based studies (Framingham cohort, Rotterdam cohort and the Atherosclerosis Risk in Communities (ARIC) study) with different definitions of gout in each of the study cohorts. It is important to notice, that the allele frequencies between Deghan et al. (rs1165205) [6], Kolz et al. [7] and our present study (both rs1183201) did not differ substantially (47%, 48% and 48%, respectively).

The same might hold true for the other SNPs that were genotyped in our present study but showed no significant association with the phenotype gout. However, one has to emphasize that in our study, we explicitly investigated the role of SNP markers with clinically manifest gout or gouty arthritis, for which elevated serum UA levels are an important but not a mandatory risk factor [21], [22]. Therefore, differences in the pathophysiological pathways of the development of elevated serum UA levels and the ignition of the inflammatory process of gout or gouty arthritis may account for the distinct findings of our study. This is also reflected by the clinical observations that many patients with high serum UA levels never experience an attack of gout, whereas other people in the absence of hyperuricemia suffer from severe and recurrent flares of gouty arthritis [22]. One can speculate that other pathophysiological mechanisms might be involved, or a complex interplay of genes and their variants lead to the manifestation of the disease. For example, the well-known URAT1 transporter, encoded by *SLC22A12* gene, is involved in renal urate exchange [23], and a *SLC22A12* polymorphism is also linked to serum UA levels [7]. Therefore, this gene is a strong candidate for gout, but does not show significant association with the clinical phenotype in this and a previous study [6]. Another possible explanation is the small effect size of some polymorphisms on serum UA levels that could directly impact susceptibility to gout. Our present study showed a high degree of association between gout and SNPs in *ABCG2* and *SLC2A9*, those polymorphisms that were reported to have highest effects on serum UA levels found in the previous meta-analysis (explaining 0.57% and 3.53% of variability, respectively) [7] and that showed ORs for gout between 1.37 and 1.52 in our study. All other SNPs were significant on a genome-wide level, but explained less the variability of serum UA levels (below 0.2%) [7]. Therefore, either power was not sufficient for detection of association between these SNPs and gout in the present study, or their relevance on the clinical phenotype gout is not evident.

Additionally, we found only weak epistatic interaction between SNPs in *SLC2A9* and *LRRC16A* on gout, making a relevant additive effect of SNPs influencing serum UA levels on the qualitative trait unlikely. Potential confounders, such as different medications and prevalence of type 2 diabetes, smoking or BMI, did not influence the association results significantly. Taken together, it is obvious that SNPs with highest influence on serum UA levels could be directly linked to susceptibility to gout, whereas the relevance of less contributing polymorphisms is still arguable.

More complex functional studies are warranted in the future to elucidate the pathways with which the newly identified genes impact serum UA levels and development of gout.

Furthermore, the presence of hyperuricemia and gout has often been discussed to be a cardiovascular risk factor [24]–[28]. We thus examined the SNPs being associated with elevated UA serum levels in our second case-control study consisting of CAD cases and controls from the general population. Here, we did not detect a direct genetic relationship between the tested SNPs and CAD. One possible explanation may be limited power: polymorphisms with a small effect on disease susceptibility require very large study samples to be detected. Therefore, we cannot rule out a causal link between the SNPs influencing serum UA levels and CAD. On the other hand, CAD is possibly a more heterogeneous disorder than gout, even on genetic level. For example, no genes known to influence serum UA levels were identified by recent GWA studies on CAD, but genetic loci involved in several different pathways were found [11], [12], [29]–[32].

There are limitations in our study design that have to be considered. First, we do not have measurement of serum UA levels in our cohort. Hence, we can not directly replicate the findings of Kolz et al. on serum UA levels [7]. However, we did not aim in replication of serum UA level association but in expansion of these results to clinical manifestation of the phenotypes gout and CAD. Second, all phenotypes were assessed retrospectively from patient documentations and medical history readings. When gout was diagnosed by a physician according to ICD-9 code 274, the phenotype gout was considered as confirmed. In case of self-reported gout, additional intake of uricostatic or uricosuric medication was required to affirm the diagnosis of gout. We have follow-up data from more than 80% of our study participants and, therefore, validation of clinicial phenotypes is available. Third, the power to analyze gender effects in our study is limited. As previously described, association of serum UA levels depends to some degree on gender [4]. Our findings on gender-specific association between male but not female gout patients and rs2231142 in *ABCG2* gene are likely to be true positive results but some other gender effects may have been overlooked. Forth, assuming that gout is a risk factor for CAD, we expected to observe significantly more CAD patients in the gout sample than in gout-free controls. However, based on our initial ascertainment strategy where we retrospectively identified gout patients and gout-free controls from a MI/CAD study cohort, we did not find a significant coincidence of CAD and gout. On the other hand, in our CAD case-control sample we found that the clinical phenotype of gout seems to be associated with CAD.

In conclusion, we performed a comprehensive analysis on association with susceptibility to gout and CAD of recently published polymorphisms known to be linked with serum UA levels. Markers in *SLC2A9* and *ABCG2* genes are strongly associated with clinical manifestation of gout in the German MI Family Study. With the knowledge of a comprehensive number of genetic polymorphisms contributing to gout, genetic testing as a supportive diagnostic tool would be conceivable.
